# Pharmaceutical, Clinical, and Regulatory Challenges of Reformulating Pressurized Metered-Dose Inhalers to Reduce Their Environmental Impact

**DOI:** 10.1089/jamp.2024.0023

**Published:** 2025-02-03

**Authors:** Nicolas Roche, Omar Usmani, Laura Franzini, Lorenza Labadini, Kusum S. Mathews, Sara Panigone, Job F.M. van Boven

**Affiliations:** ^1^ Service de Pneumologie, Hôpital Cochin, APHP Centre et Université Paris Cité, Institut Cochin, INSERM UMR 1016, Paris, France.; ^2^ Inserm, Équipe d’Épidémiologie Respiratoire Intégrative, CESP, Villejuif, France.; ^3^ NHLI Imperial College London, London, United Kingdom.; ^4^ Chiesi Farmaceutici SpA, Parma, Italy.; ^5^ Department of Clinical Pharmacy and Pharmacology, University Medical Center Groningen, Groningen Research Institute for Asthma and COPD (GRIAC), University of Groningen, Groningen, The Netherlands.; ^6^ Medication Adherence Expertise Center of the northern Netherlands (MAECON), Groningen, The Netherlands.; ^7^ Centre for Medicine Use and Safety, Monash Institute of Pharmaceutical Sciences, Monash University, Melbourne, Australia.

**Keywords:** global warming, hydrofluoroalkanes, inhaler, ozone depletion

## Abstract

The chlorofluorocarbons (CFCs) that were used as propellants in early pressurized metered-dose inhalers (pMDIs) had substantial ozone-depleting potential. Following the Montreal Protocol in 1987, the manufacture of a range of ozone-depleting substances, including CFCs, was gradually phased out, which required the propellants used in pMDIs to be replaced. Current pMDIs use hydrofluoroalkanes (HFAs) as propellants, such as 1,1,1,2-tetrafluoroethane (HFA-134a). Although these HFAs have no ozone-depleting potential, they have a high global warming potential (GWP), and consequently, their use is being phased down. One option for the discontinuation of HFA use in inhalers would be to discontinue all pMDIs, switching patients to dry powder inhalers (DPIs). However, a switch from pMDIs to DPIs may not be a clinically appropriate option for some patients; furthermore, the full lifecycle carbon footprint and the overall environmental impact of different inhalers should be considered. An alternative is therefore to reformulate the current HFA pMDIs to use low-GWP propellants, such as 1,1-difluoroethane (HFA-152a). This article summarizes the various steps and challenges associated with this change, illustrated using data from the inhaled triple combination of beclomethasone dipropionate, formoterol fumarate, and glycopyrronium bromide, a complex formulation of three molecules in a solution that contains liquid-phase propellant.

## Introduction

Pressurized metered-dose inhalers (pMDIs) are the most widely used inhaler type globally, with active pharmaceutical ingredients either dissolved or suspended in liquid propellant. Historically, chlorofluorocarbons (CFCs) were used as propellants, yet they have substantial ozone-depleting potential.^1^ Following ratification of the Montreal Protocol in 1987,^2^ the manufacture of a range of ozone-depleting substances, including CFCs, was gradually phased out, which required other propellants to be used in pMDIs.

Switching the propellant is not a straightforward process, especially given the regulatory requirement to demonstrate at least equivalent safety and efficacy following any reformulation.^3^ As a consequence, pMDIs received an essential use exemption that permitted the continuation of CFC use until alternatives were developed, tested, and available. Indeed, it took until 1995 for non-CFC pMDIs to be launched. These use hydrofluoroalkanes (HFAs) as propellants, such as 1,1,1,2-tetrafluoroethane (HFA-134a) and 1,1,1,2,3,3,3-heptafluoropropane (HFA-227ea), both of which are fluorinated greenhouse gases (or F-gases).

Although HFAs have no ozone-depleting potential, many have a high global warming potential (GWP)—for example, HFA-134a has a GWP 1300 times that of carbon dioxide.^1^ The European Union (EU) passed a regulation in 2014 that aimed to protect the environment by reducing F-gas emissions, although exempting the pMDI sector.^4^ In 2016, the Kigali Amendment to the Montreal Protocol was signed to control the production and consumption of hydrofluorocarbons (HFCs, which includes HFAs), encouraging the use of low-GWP HFC alternatives to minimize emissions.^5^ Furthermore, the EU revised their F-gas regulations to include the medical sector from March 2024, incorporating a phase-down of the use of HFCs in pMDIs that provides sufficient time to develop alternative gases and so avoid treatment shortages.^6^

This article provides an overview of the numerous pharmaceutical, clinical, and regulatory challenges that need to be considered when reformulating pMDIs to use low GWP propellants, with a focus on the steps and associated challenges.

### Considering the full carbon footprint lifecycle

While one option for lowering the impact of inhalers on the environment is reformulation to lower GWP propellants, another would be to switch all patients from pMDIs to alternative inhaler devices, that is, dry powder inhalers (DPIs) and soft mist inhalers. Indeed, this was the initial approach advocated by the National Health Service in England.^7^

**FIG. 1.**
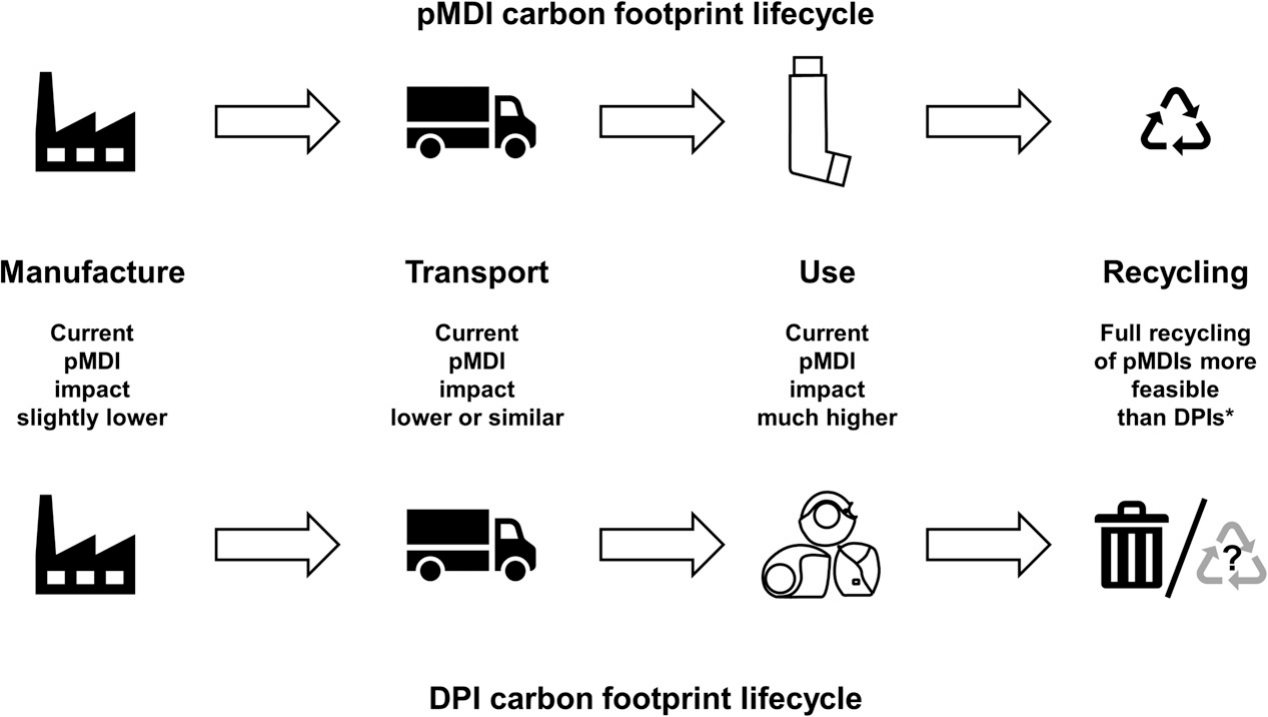
Relative carbon footprint lifecycles of pMDIs and DPIs (based on information in Panigone et al.^9^ and Murphy et al.^10^). *The feasibility and environmental effectiveness of a postal inhaler recovery and recycling scheme was evaluated in the UK in 2021 and 2022. All components of returned pMDIs were recycled; DPIs were incinerated to produce energy-from-waste.^10^ DPIs, dry-powder inhaler; pMDI, pressurized metered-dose inhaler.

When considering the overall environmental impact of an inhaler, it is important to take into account not only the GWP of gases emitted when they are used but also their overall lifecycle carbon footprint, together with other environmental impacts related to the sourcing and purification of raw materials, energy production, manufacture, transport, and disposal upon emptying.^8^ Although pMDIs have higher emissions than DPIs at the point of use, other points of their overall lifecycle, such as their manufacture and transport, may incur a slightly lower carbon footprint (Fig. 1).^9^ Indeed, in a study assessing broader lifecycle environmental impacts, although pMDIs had a higher impact in terms of GWP, DPIs tended to have a higher impact on other dimensions such as fossil fuel depletion and marine ecotoxicity.^8^ However, the relative carbon footprint of device types will depend on the complexity of the devices and of their production processes and can vary from country-to-country depending on, for example, the type of energy used for manufacturing (fossil fuels or renewable sources). Furthermore, a recovery and recycling project has shown pMDIs are easier to recycle (partly due to the wide variability in DPI designs),^10^ although the ease of recycling will depend on the availability of (and regulations around) effective waste management and recycling programs that can accommodate the mixture of components in an inhaler, including the mixture of plastic polymers in many DPIs. Overall, therefore, the wide range of factors that impact the carbon footprint of devices (and their variability) make it difficult for physicians and patients to decide which inhaler type is most environmentally friendly.

### Considerations when switching inhalers

Switching inhaler devices also involves several complex considerations beyond the environmental aspects, including the pharmaceutical agent and clinical effect.^11^ Most switches from pMDI to DPI are associated with a change of active molecule(s)—which may be a concern in a patient whose disease is well controlled. In addition, key factors in the choice of inhaled medication include the patient’s ability to use the inhaler device correctly and their device preference, both of which are known determinants of adherence.^12,13^ Therefore, it is of paramount importance that any inhaler device choice is personalized.^14,15^ Indeed, although DPIs may be preferred over pMDIs for some patients, such as those who cannot coordinate actuation with inhalation, they are not suitable for all patients. For example, DPIs would not be suitable for the very young, who typically receive inhaled medication from a pMDI plus spacer or facemask combination (the manufacturing and use of which would need to be taken into account when considering the overall carbon footprint of pMDI use). Furthermore, some patients are not able to generate the minimum inspiratory force required to aerosolize the powder with some DPIs or are unable to inhale a sufficient volume (not receiving the correct dose in either case),^16–18^ although this may be less of an issue with the newer devices, many of which have higher resistance.^19^ Even when a switch from pMDI to DPI is discussed (and agreed) with the patient and appropriate training and support is provided, asthma control and chronic obstructive pulmonary disease (COPD) health status can be substantially impacted by the switch, despite high overall satisfaction.^20^ The potential impact of a multiple-inhaler regimen must also be considered—since many patients use a short-acting β_2_-agonist pMDI as their rescue medication, the use of a DPI to deliver maintenance therapy increases the risk of use errors and resulting adverse effects on asthma and COPD outcomes (e.g., decreased symptom control and higher exacerbation rates).^21–23^ If changes in a patient’s health result in hospitalization, this can induce substantial greenhouse gas emissions, together with other environmental waste such as solid plastics, especially if the patient requires intensive care.^24,25^ Furthermore, beyond the clinical and environmental costs of loss in disease control, these “nonclinical” switches can have both financial and nonfinancial implications, with one study estimating overall additional resource requirements if all patients are switched from pMDIs to DPIs of up to £60 million for the English National Health Service when staff counseling costs are taken into consideration.^26^ The switch can also have a negative impact on the doctor–patient relationship, especially if the switch is nonconsented, where the involuntary nature of the change in inhaler can leave the patient angry, upset, or shocked.^11,27^

### Considerations when reformulating a pMDI

Whereas a switch from a pMDI to a DPI puts the burden on healthcare teams and patients, reformulating a pMDI to use a new propellant (such that the patient can continue to use a familiar device containing the same active ingredients) puts the burden on the manufacturer while maintaining patient options. This is the long-term approach taken by a number of pharmaceutical companies, which aim to reformulate pMDIs to use a low-GWP propellant in place of the currently used HFAs. This is not a straightforward process as the active ingredient (i.e., the molecule that has the pharmacological effect) is in solution or suspension in the propellant within the pMDI canister, which means that the propellant is an excipient. As a consequence, changing the propellant potentially impacts the whole formulation and how that formulation performs.

**FIG. 2.**
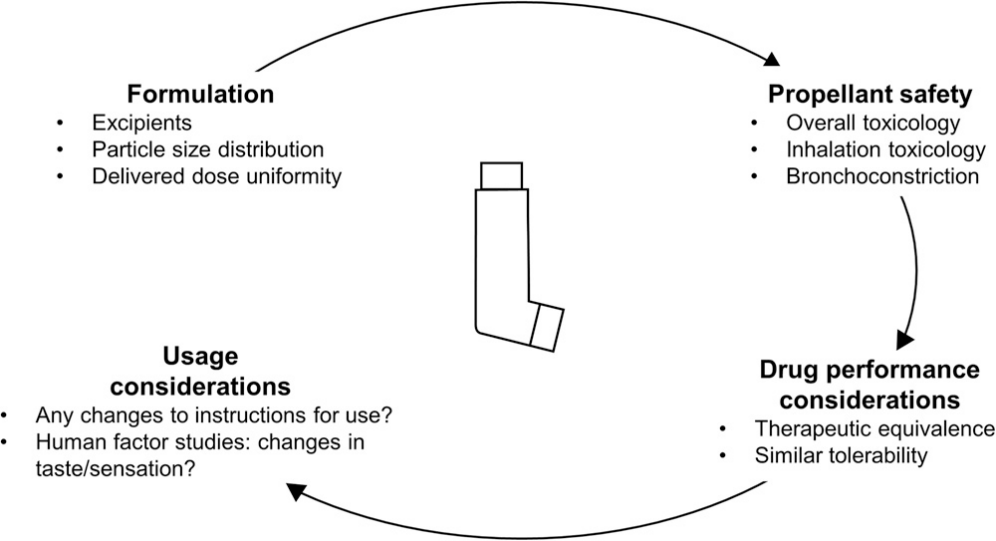
Key considerations when reformulating a pMDI.

Key considerations for this reformulation are shown in Figure 2 and are discussed in more detail in the following sections. The four steps in this process (formulation, propellant safety, drug performance, and usage considerations) are based on European Medicines Agency (EMA) regulatory requirements, as this organization has produced specific guidance^28^; specific requirements for other regulators may vary slightly, although the four broad areas are likely to apply to all. Two low-GWP propellants are currently under development: 1,1-difluoroethane (HFA-152a) and 1,3,3,3-tetrafluoropropene (HFO1234ze).^29^ Different pharmaceutical companies are studying either one or both of the propellants in their pMDI reformulation work, based on considerations linked with the need to retain the clinical properties of treatments being developed, together with existing safety data.^30^ For example, HFA-152a has extensive safety data for human use, including a propellant-only clinical trial (see “Propellant Safety” section). For consistency and focus, the considerations in this article are illustrated by data from the reformulation work being done by Chiesi Farmaceutici SpA, with a focus on the inhaled triple therapy combination of beclomethasone dipropionate (BDP), formoterol fumarate (FF), and glycopyrronium bromide (GB).

## Formulation

The propellant selected to replace HFA-134a in Chiesi’s pMDIs is HFA-152a (Koura, Cheshire, UK), which has a GWP 138 times that of carbon dioxide—approximately 11% of HFA-134a.^1^ An aim of the HFA-152a formulation was to develop a pharmaceutically comparable product with the same inhalation performance and quality profile as the HFA-134a formulation, taking into account the different physical properties of HFA-152a. HFA-152a has similar physicochemical properties to HFA-134a in terms of boiling point and density.^31^ In addition, full *in vitro* characterization of the BDP/FF/GB HFA-152a formulation is needed to confirm that the aerodynamic particle size distribution, delivered dose uniformity, and characteristics of the emitted plume are comparable with the HFA-134a formulation. This BDP/FF/GB reformulation has followed an innovative approach in which *in silico* mathematical modeling was used to predict *in vitro* performance, followed by validation of the aerodynamic particle size distribution using standard compendial methods.

## Propellant Safety

Unlike HFA-134a, HFA-152a is technically classified as a flammable substance.^31^ However, detailed risk assessments of pMDIs containing limited volumes of HFA-152a, including flammability tests, suggest that the final formulation is not expected to have an impact on patient safety.^32^ The flammability of the new propellant has mainly required changes to the manufacturing process, and a new manufacturing site has been constructed for HFA-152a-containing pMDIs, specifically designed for the management of high volumes of flammable substances.

### Overall and inhalation toxicology

HFA-152a is commonly used in consumer aerosols and so has well-characterized toxicology (similar to that of HFA-134a). However, manufacturers are required to develop a full regulatory toxicology package for propellants used in pharmaceutical products. In a series of animal inhalation studies, HFA-152a was well tolerated and showed no acute or chronic toxicity, no genotoxic or carcinogenic activity, no developmental or reproductive toxicity, and no cardiopulmonary or respiratory toxicity.^31,33^ Subsequently, a human toxicology Phase I study was conducted to evaluate the safety, tolerability, taste, and pharmacokinetics of orally inhaled HFA-152a in eight healthy adult males. Overall, HFA-152a was well-tolerated, had minimal impact on the main aspects of taste scoring, and was rapidly cleared from the blood.^31^

### Bronchoconstriction

Anything that is inhaled has the potential to trigger bronchoconstriction. In addition to human toxicology, a study was therefore conducted in patients with asthma to evaluate the bronchoconstriction potential of HFA-152 by comparing the change from baseline in forced expiratory volume in 1 second (FEV_1_) at 15 minutes postdose following inhalation of HFA-152a and HFA-134a.^34^ There were no bronchoconstriction events, and equivalence of the two formulations was demonstrated. The clinical safety of the propellant alone and of the final product formulation has been or is being further investigated in a series of studies, including a study on the effect of HFA-152a on normal lung mucociliary clearance (ClinicalTrials.gov registration numbers NCT05875025 and NCT06264674).

## Drug Performance Considerations

### Therapeutic equivalence

**FIG. 3.**
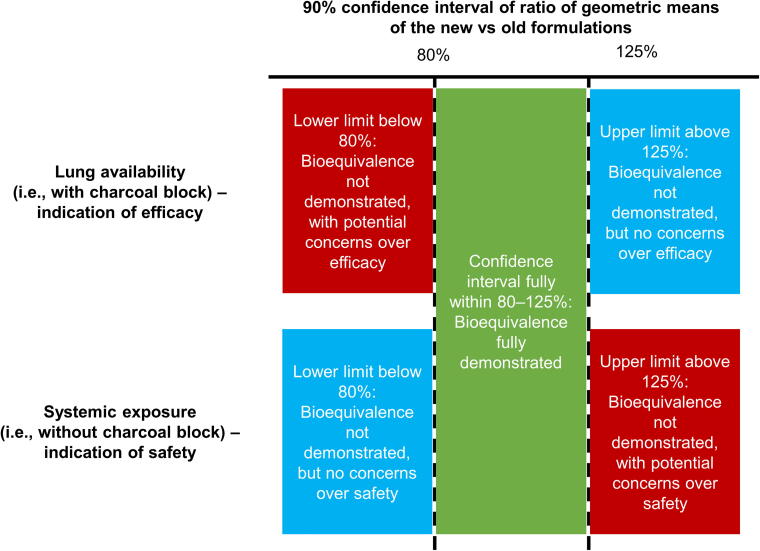
European Medicines Agency bioequivalence criteria,^35^ applied to inhaled formulations.

In this section, we discuss the demonstration of pharmacokinetic bioequivalence of two inhaled formulations based on the EMA bioequivalence criteria—and specifically that the 90% confidence interval (CI) of the ratio of geometric means for the constituents of the new (HFA-152a) versus reference (HFA-134a) formulations should be contained between 80% and 125%.^35^ These bioequivalence criteria should be met following pharmacokinetic comparisons that are performed (1) with a charcoal block (i.e., subjects consume a charcoal solution prior to and after inhalation), to evaluate lung availability as a marker of efficacy, and then (2) without a charcoal block, to evaluate systemic exposure as a marker of safety. As illustrated in Figure 3, in the charcoal block evaluation, a lower CI limit <80% indicates that the efficacy of the new HFA-152a formulation may be lower than that of the current HFA-134a formulation. In this case, regulatory authorities may request follow-on pharmacodynamic equivalence studies to support therapeutic equivalence. If the upper CI limit is >125%, there are no concerns regarding efficacy, even though bioequivalence has not been formally demonstrated. In contrast, in the evaluation without charcoal block, if the upper CI limit is >125%, there is a potential safety signal, and regulatory authorities may request follow-on studies. If the lower CI limit is <80%, safety concerns are unlikely, even though bioequivalence has not been formally demonstrated.

To evaluate the pharmacokinetic bioequivalence of the BDP/FF/GB HFA-152a and HFA-134a formulations, three studies were conducted, each in approximately 70 healthy volunteers.^36^ In Studies 1 and 2, subjects inhaled four puffs of BDP/FF/GB (Study 1: 100/6/12.5 µg [medium-strength BDP]; Study 2: 200/6/12.5 µg [high-strength BDP]), ingesting activated charcoal in two of the periods (once per propellant). Study 3 provided supportive data by administering medium- and high-strength BDP/FF/GB via the AeroChamber Plus spacer. Note that only the BDP dose differs between medium- and high-strength BDP/FF/GB.

The two key pharmacokinetic parameters in Studies 1 and 2 were the area under the plasma concentration—time curve between time zero and the last quantifiable timepoint (AUC_0–_*_t_*) and the maximum plasma concentration (*C*_max_). In addition to evaluating the pharmacokinetics of BDP, formoterol, and GB, these studies also evaluated the pharmacokinetics of beclomethasone-17-monopropionate (B17MP), the active metabolite of BDP. In Study 3, the key outcomes were AUC_0–_*_t_*, *C*_max_, and area under the plasma concentration—time curve from time zero to 30 minutes (AUC_0–30 min_).

**FIG. 4.**
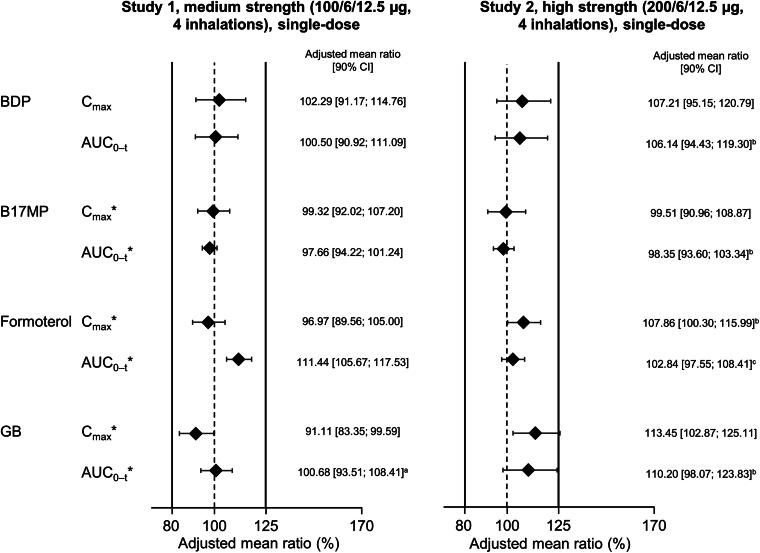
Relative total systemic exposure following administration of BDP/FF/GB via pMDI with HFA-152a propellant vs. HFA-134a, both administered without charcoal block (Reprinted from Rony et al. Pulm Pharmacol Ther 2024;102299, copyright 2024, with permission from Elsevier).^36^ *Indicates a primary endpoint. The solid vertical lines at 80% and 125% indicate the bioequivalence criteria. *n* = 59 for Study 1 and 62 for Study 2, except ^a^58; ^b^61; and ^c^60. The adjusted mean ratio is the ratio of adjusted geometric means of log-transformed data. AUC_0–_*_t_*, area under the plasma concentration—time curve between time zero and the last quantifiable timepoint; B17MP, beclomethasone 17 monopropionate; BDP, beclomethasone dipropionate; *C*_max_, maximum plasma concentration; FF, formoterol fumarate; GB, glycopyrronium bromide; pMDI, pressurized metered-dose inhaler.

**FIG. 5.**
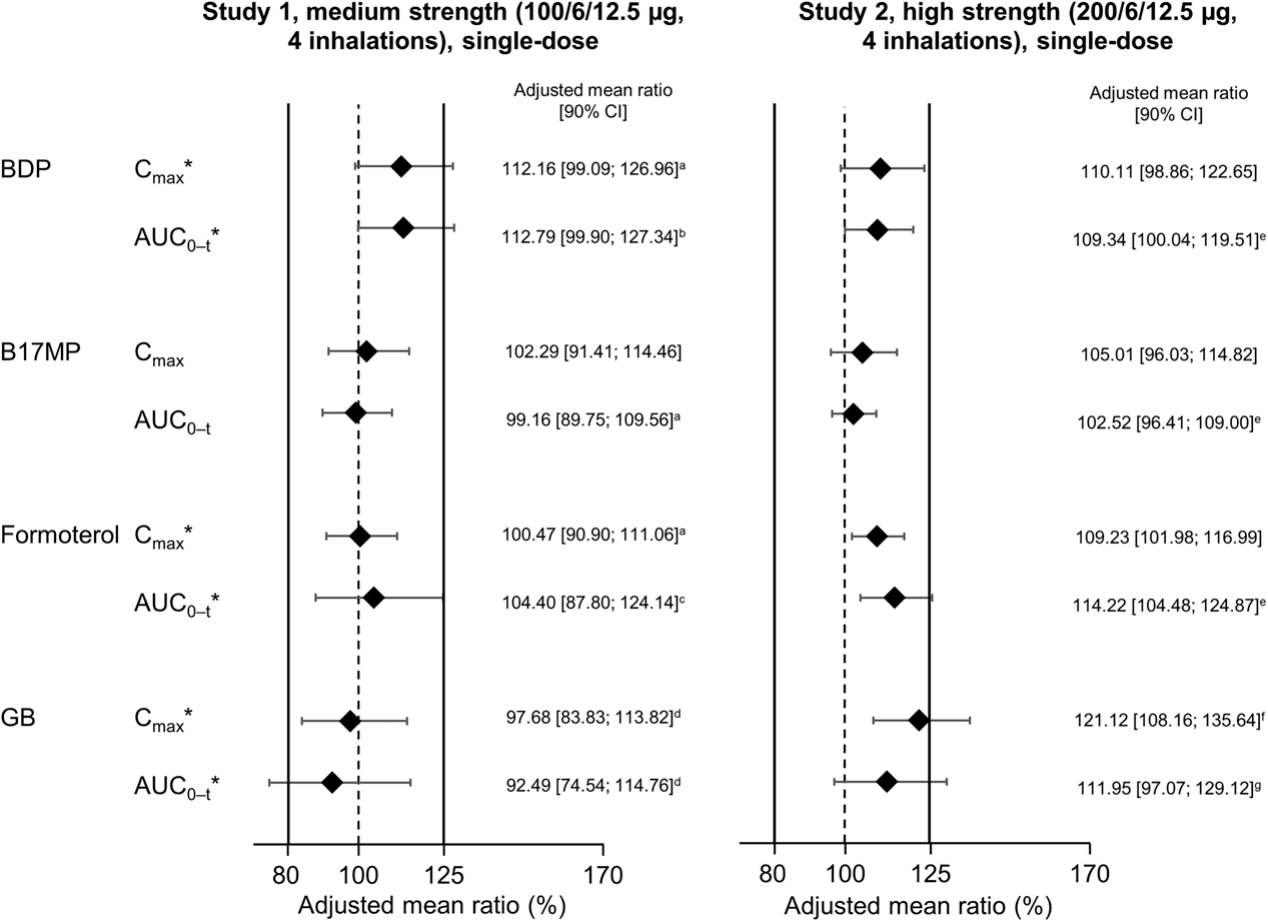
Relative lung availability following administration of BDP/FF/GB via pMDI with HFA-152a propellant vs. HFA-134a, both administered with charcoal block (Reprinted from Rony et al. Pulm Pharmacol Ther 2024;102299, copyright 2024, with permission from Elsevier).^36^ *Indicates a primary endpoint. The solid vertical lines at 80% and 125% indicate the bioequivalence criteria. *n* = 59 for Study 1 and 63 for Study 2, except ^a^58; ^b^57; ^c^56; ^d^55; ^e^62; ^f^61; and ^g^59. The adjusted mean ratio is the ratio of adjusted geometric means of log-transformed data. AUC_0–t_, area under the plasma concentration—time curve between time zero and the last quantifiable timepoint; B17MP, beclomethasone 17 monopropionate; BDP, beclomethasone dipropionate; C_max_, maximum plasma concentration; FF, formoterol fumarate; GB, glycopyrronium bromide; pMDI, pressurized metered-dose inhaler.

In terms of safety (i.e., systemic exposure and without charcoal block), bioequivalence was demonstrated for all four analytes in Study 1 and for BDP, B17MP, and formoterol in Study 2 (Fig. 4). For GB in Study 2, the upper 90% CI limit for the *C*_max_ analysis marginally exceeded the bioequivalence limit by 0.11%. This is unlikely to indicate a safety concern. In the lung availability comparisons (i.e., with charcoal block), B17MP and formoterol met the equivalence criteria in both Study 1 and 2 (Fig. 5). The criteria were also met for BDP in Study 2, but the upper limits were exceeded in Study 1 (i.e., following administration of medium strength). The upper limits were also exceeded for GB in Study 2. Given lung availability is a marker of efficacy, these two results are unlikely to indicate a concern. However, in Study 1, the GB AUC_0–_*_t_* lower limit was exceeded (i.e., was <80%). In a *post hoc* evaluation, it appeared that two of the subjects incorrectly administered the medium-strength test drug, and when their data were excluded, the equivalence criteria were met.

**FIG. 6.**
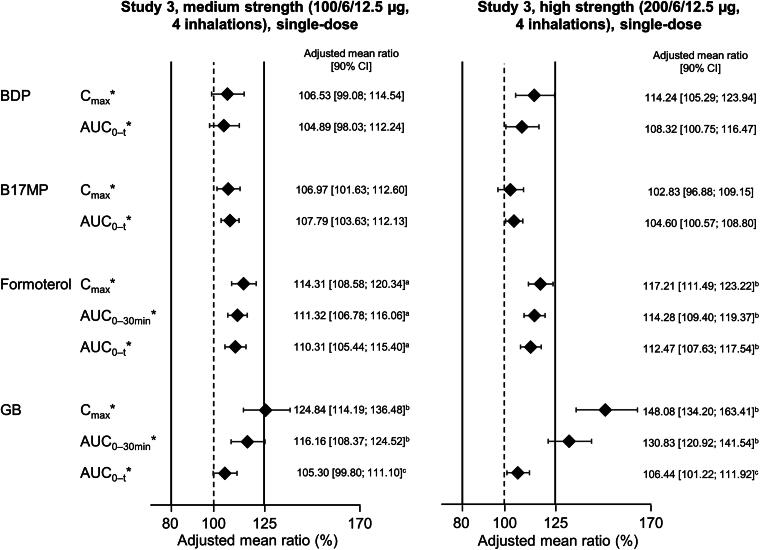
Comparison of primary pharmacokinetic parameters following administration of BDP/FF/GB via pMDI with HFA-152a propellant vs. HFA-134a, both via spacer (Reprinted from Rony et al. Pulm Pharmacol Ther 2024;102299, copyright 2024, with permission from Elsevier).^36^ *Indicates a primary endpoint. The solid vertical lines at 80% and 125% indicate the bioequivalence criteria. *n* = 67 for medium strength and 66 for high strength, except ^a^65; ^b^64; and ^c^63. The adjusted mean ratio is the ratio of adjusted geometric means of log-transformed data. AUC_0–t_, area under the plasma concentration—time curve between time zero and the last quantifiable timepoint; AUC_0-30min_, area under the plasma concentration—time curve from time zero to 30 min; B17MP, beclomethasone 17 monopropionate; BDP, beclomethasone dipropionate; C_max_, maximum plasma concentration; FF, formoterol fumarate; GB, glycopyrronium bromide; pMDI, pressurized metered-dose inhaler.

In Study 3 (in which BDP/FF/GB was administered using a spacer), BDP, B17MP, and formoterol all met the bioequivalence criteria after administration of both medium- and high-strength BDP/FF/GB. Systemic availability of GB, assessed using *C*_max_, was higher with the HFA-152a formulation; however, the bioequivalence criteria were met for AUC_0–_*_t_* (Fig. 6). For AUC_0–30min_, which evaluates lung availability, bioequivalence was met for one of the comparisons (100/6/12.5 µg) but not the other (200/6/12.5 µg), and it should be noted that both BDP/FF/GB strengths contain the same amount of GB. Overall, therefore, while formal bioequivalence cannot be concluded for all analytes, these data suggest comparability of the new formulation with the existing BDP/FF/GB pMDI, thereby supporting the reformulation. The same process is being followed for other products in Chiesi’s pMDI portfolio, including an inhaled corticosteroid/long-acting β_2_-agonist (ICS/LABA) combination and a single-agent ICS.

### Tolerability

In addition to evaluating the likelihood of the propellant to induce bronchoconstriction (see “Propellant Safety” section), the overall tolerability of the HFA-152a formulation of BDP/FF/GB was compared with that of the HFA-134a formulation in the pharmacokinetic studies.^36^ Of note, subjects inhaled double the licensed dose in these studies. The overall incidence of adverse effects was similar with the two formulations, with the majority of events not related to study treatment. The only events to occur in two or more subjects with any treatment were headache and tremor, both being known β_2_-agonist dose-related adverse effects. The EMA has requested additional 3-month safety data; this study is ongoing at the time of writing (ClinicalTrials.gov registration number NCT06264674).

## Usage Considerations

### Instructions for use

A key advantage for retaining pMDIs in the panel of available inhaler devices is that, given their wide use globally, this device type is familiar to patients and uniform instructions can be provided across a range of pharmaceutical maintenance and reliever ingredients (whereas each DPI has slightly different instructions for use). A change in propellant is unlikely to change the way that a pMDI is used and is therefore unlikely to impact the instructions for use.

### Human factor studies

A key aspect of inhaler use is the experience of patients with the inhaler devices. One issue with the historic switch from CFC to HFA propellants was a change in the sensation at the back of the throat following dose emission. CFC propellants caused the so-called “cold freon effect” when the plume impacted the back of a patient’s throat,^3^ which caused some patients to stop inhaling (and so receive a suboptimal dose). HFAs produce warmer plumes than CFCs,^37^ meaning that HFA-134a does not have this cold freon effect. In the Phase I study, 6 of the 8 participants reported a slight cold sensation immediately following inhalation of HFA-152a, but the average score was 1.4 out of 10, indicating that this was not considered a clinical concern.^31^ More detailed human factor studies are planned, with the aim of confirming that the reformulation does not affect patient usability, with any possible minor variations reflected in the instructions for use.

## Discussion

In this article, we have summarized the various challenges associated with pMDI reformulation from a development, implementation, and use perspective, focusing on the inhaled triple therapy BDP/FF/GB. This is a complex formulation given the three molecules are dissolved in a liquid-phase propellant. Thus, the replacement of the propellant has the potential to impact the delivery of one or several of the three molecules. Other products, such as ICS/LABA combinations and ICS monotherapy, will capitalize on this work, with the transition to HFA-152a anticipated to reduce the carbon footprint of pMDIs by up to 90%, to a level similar to that of DPIs.^9^

Overall, the goal of reformulating pMDIs to use low-GWP propellants has been to make the experience of the propellant switch seamless for patients and to retain their therapeutic options while lowering the impact in terms of greenhouse gas emissions.^38^ When switching from HFA-134a to HFA-152a, there are no changes to taste or sensation (unlike with the historic switch from CFC to HFA propellants, where there was a change in patient perception of the “cold freon” effect), and the instructions for use are unlikely to change (as would be the case for a switch from pMDI to DPI). Furthermore, the BDP/FF/GB bioequivalence and particle size distribution data confirm the validity of the reformulation work and suggest that there will be no need to alter the dose for either efficacy or safety considerations. This is important, as in a large global survey spanning 42 countries and six continents that assessed inhaler choice and optimized sustainable health care, both healthcare practitioners (*N* = 468) and patients with asthma or COPD (*N* = 270) ranked therapeutic efficacy (72.2%) and preventing future health deterioration (80.7%) as the highest priorities when choosing an inhaler, whereas fewer than half (45.9%) of healthcare practitioners and less than two thirds (60.1%) of patients were concerned about potential inhaler contribution to climate change.^39^

Another reason that the pMDI reformulation is essential is that under the EU REACH (Registration, Evaluation, Authorisation and Restriction of Chemicals) regulation, five countries have proposed restricting the use of a number of substances defined as per- and polyfluoroalkyl substances (PFAS), which persist in the environment and can, in some cases, have harmful effects on people, plants, and animals.^40^ In contrast to propellants currently used in pMDIs and to HFO1234ze, HFA-152a is not classified as a PFAS according to the European Chemicals Agency.^40^

In a broader environmental impact reduction context, reformulation of pMDIs could be complimented by other waste-reduction strategies, such as recycling programs (where feasible and appropriate),^10^ combining multiple single inhalers into one inhaler where possible,^41^ promoting inhaler adherence,^42^ and limiting short-acting β_2_-agonist overuse,^43^ while ensuring continued global access to inhalers.^44^

For pMDIs, the majority of the carbon footprint depends on the propellant. Although pMDIs currently account for a limited proportion of the total global greenhouse gas emissions, as other sectors transition to low GWP options, this proportion is set to increase. Timely action is therefore needed in the medical sector, while also safeguarding treatment efficacy and safety for patients. In addition, propellant reformulation should be considered the first step in reducing the overall impact of such therapies, and other aspects of the carbon footprint (such as the impact of manufacturing and transport) should be addressed by manufacturers, for example, by transitioning from fossil fuel-based energy to renewable sources. Furthermore, organizations such as the European Commission are developing plastics strategies that are likely to transform the way plastic products are designed, produced, used, and recycled.^45^

## Conclusions

Given the need to phase down use of propellants with high GWP, it is important to reformulate pMDIs to use propellants with low GWP, such as HFA-152a. This requires a consideration of a range of factors, including propellant safety and the impact on patient use, ideally making the experience for the patient seamless. In particular, the new formulation should demonstrate bioequivalence, such that no change in dose is required. Demonstrating bioequivalence is especially complex when the pMDI delivers combinations of active ingredients, such as BDP/FF/GB. The work summarized in this article will inform the reformulation of a wide range of pharmacological compounds for delivery via pMDI with this new propellant.

## Abbreviations Used

AUC_0–30min_: area under the plasma concentration–time curve from time zero to 30 min
AUC_0–t_: area under the plasma concentration–time curve between time zero and the last quantifiable timepoin74
B17MP: beclomethasone-17-monopropionate
BDP: beclomethasone dipropionate
C_max_: maximum plasma concentration
CFC: chlorofluorocarbon
CI: confidence interval
COPD: chronic obstructive pulmonary disease
DPI: dry powder inhaler
EMA: European Medicines Agency
EU: European Union
F-gas: fluorinated greenhouse gas
FEV_1_: forced expiratory volume in 1 second
FF: formoterol fumarate
GB: glycopyrronium bromide
GWP: global warming potential
HFA-134a: 1,1,1,2-tetrafluoroethane
HFA-152a: 1,1-difluoroethane
HFA-227ea: 1,1,1,2,3,3,3 heptafluoropropane
HFC: hydrofluorocarbon
HFO1234ze: 1,3,3,3-tetrafluoropropene
ICS: inhaled corticosteroid
LABA: long-acting β_2_-agonist
pMDI: pressurized metered-dose inhaler
